# Subverting ER-Stress towards Apoptosis by Nelfinavir and Curcumin Coexposure Augments Docetaxel Efficacy in Castration Resistant Prostate Cancer Cells

**DOI:** 10.1371/journal.pone.0103109

**Published:** 2014-08-14

**Authors:** Aditi Mathur, Zakaria Y. Abd Elmageed, Xichun Liu, Mikhail L. Kostochka, Haitao Zhang, Asim B. Abdel-Mageed, Debasis Mondal

**Affiliations:** 1 Department of Pharmacology, Tulane University School of Medicine, New Orleans, Louisiana, United States of America; 2 Department of Urology, Tulane University School of Medicine, New Orleans, Louisiana, United States of America; 3 Department of Pathology, Tulane University School of Medicine, New Orleans, Louisiana, United States of America; 4 Peptide Research Laboratories, Tulane University School of Medicine, New Orleans, Louisiana, United States of America; UC Davis Comprehensive Cancer Center, United States of America

## Abstract

Despite its side-effects, docetaxel (DTX) remains a first-line treatment against castration resistant prostate cancer (CRPC). Therefore, strategies to increase its anti-tumor efficacy and decrease its side effects are critically needed. Targeting of the constitutive endoplasmic reticulum (ER) stress in cancer cells is being investigated as a chemosensitization approach. We hypothesized that the simultaneous induction of ER-stress and suppression of PI3K/AKT survival pathway will be a more effective approach. In a CRPC cell line, C4-2B, we observed significant (p<0.005) enhancement of DTX-induced cytotoxicity following coexposure to thapsigargin and an AKT-inhibitor. However, since these two agents are not clinically approved, we investigated whether a combination of nelfinavir (NFR) and curcumin (CUR), known to target both these metabolic pathways, can similarly increase DTX cytotoxicity in CRPC cells. Within 24 hrs post-exposure to physiologic concentrations of NFR (5 µM) and CUR (5 µM) a significantly (p<0.005) enhanced cytotoxicity was evident with low concentration of DTX (10 nM). This 3-drug combination rapidly increased apoptosis in aggressive C4-2B cells, but not in RWPE-1 cells or in primary prostate epithelial cells (PrEC). Comparative molecular studies revealed that this 3-drug combination caused a more pronounced suppression of phosphorylated-AKT and higher induction in phosphorylated-eIF2α in C4-2B cells, as compared to RWPE-1 cells. Acute exposure (3–9 hrs) to this 3-drug combination intensified ER-stress induced pro-apoptotic markers, i.e. ATF4, CHOP, and TRIB3. At much lower concentrations, chronic (3 wks) exposures to these three agents drastically reduced colony forming units (CFU) by C4-2B cells. *In vivo* studies using mice containing C4-2B tumor xenografts showed significant (p<0.05) enhancement of DTX’s (10 mg/kg) anti-tumor efficacy following coexposure to NFR (20 mg/kg) & CUR (100 mg/kg). Immunohistochemical (IHC) analyses of tumor sections indicated decreased Ki-67 staining and increased TUNEL intensity in mice exposed to the 3-drug combination. Therefore, subverting ER-stress towards apoptosis using adjuvant therapy with NFR and CUR can chemosensitize the CRPC cells to DTX therapy.

## Introduction

Prostate cancer (PCa) is the second leading cause of cancer-related deaths in men in the United States. Initial treatment of localized tumors consists of surgery and radiation, followed by androgen deprivation therapy (ADT). However, ADT is only effective for an average of 18–24 months, and the recurrence of castration resistant prostate cancer (CRPC) dictates morbidity and mortality in patients [1]. Although the newer and more potent androgen receptor (AR) antagonists, e.g. MDV-3100 (enzalutamide), have shown some promise, resistance is already being encountered in the clinic [2]. Therefore, chemotherapy with taxanes remains the drug of choice for patients with aggressive and metastatic CRPCs. However, a safe and effective strategy to augment the efficacy of taxanes represents an unmet clinical need.

Docetaxel (DTX), an anti-microtubule agent, was approved by the US FDA as the mainstay treatment against CRPC [3]. Although initially effective, DTX-based regimen has only shown a median survival of 18–20 months and response rate of only 50%. Additionally, DTX exhibits significant adverse effects in patients with comorbid conditions, which mandate dose reduction which increases the possibility of selection for resistant clones. Recent studies have shown that resistance development following long-term treatment with DTX can occur due to the upregulation of PI3K/AKT signaling in CRPC cells [4], [5]. Therefore, downregulation of PI3K/AKT signaling in CRPC cells should augment the efficacy of this chemotherapeutic agent [6].

Aggressive cancer cells are also capable of escaping chemotherapy by modulating master regulatory pathways which dictate their survival or death decision making abilities. In this respect, control of protein translation via the exquisitely regulated ER-stress cascade has been shown to promote tumor cell survival and escape from apoptosis [7]. A direct link between aggressive tumor phenotype and increased expression of the ER-stress marker, BiP/Grp78, has been documented [8]–[10]. Indeed, several recent reports have established that ER-stress can facilitate persistent tumor growth and their therapeutic resistance. Therefore, investigators have suggested that the targeting of ER-stress may be a potent chemosensitizing strategy [11]–[13]. Wu et al, (2009) demonstrated that the ER-stress inducer methylseleninic acid (MSA) sensitizes PC-3 cells to the cytotoxic effects of paclitaxel and DTX [11]. Natural compounds like epigallocatechin gallate, a polyphenolic compound in green tea, can enhance chemotherapy efficacy in glioblastoma cells by increasing ER-stress [14]. However, the efficacy of simultaneous down-regulation of the PI3K/AKT survival pathway and upregulation of the ER-stress induced apoptosis as a potent chemosensitization approach has not been tested.

**Figure 1:**
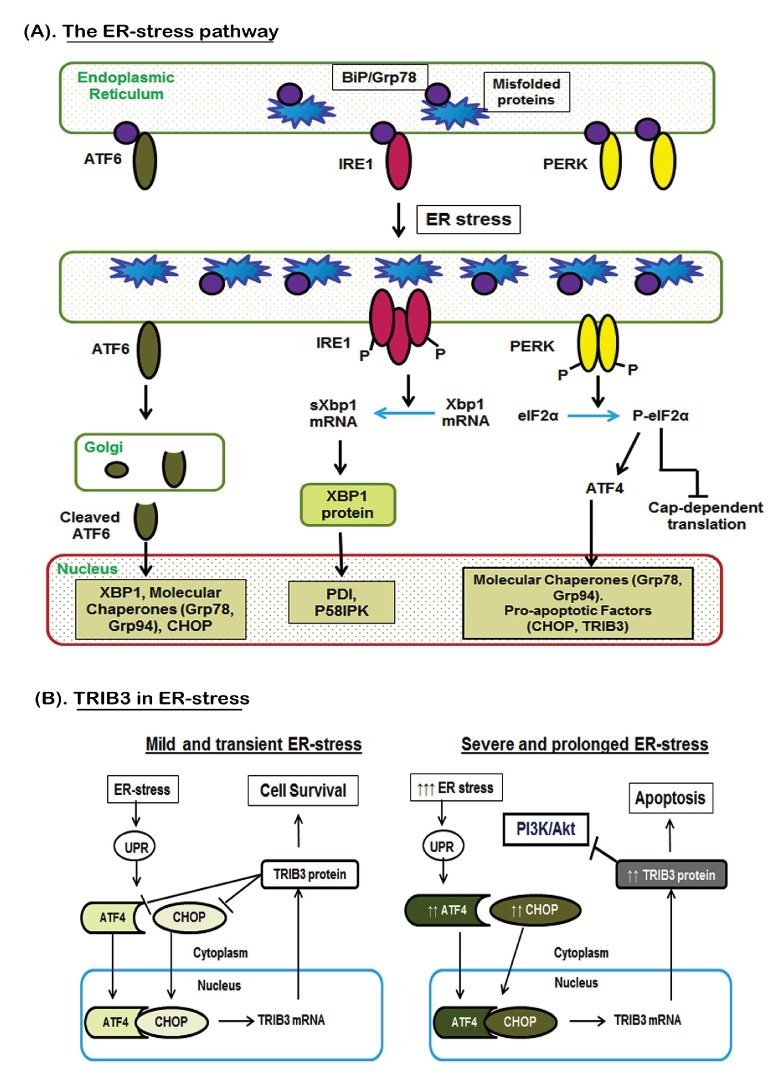
Cross-talks between ER-stress signaling pathways in dictating cell survival vs. cell death. (A). Under normal homeostatic conditions, the ATF6, IRE1 and PERK proteins are bound to BiP/Grp78 at the ER membrane. An unfolded protein response (UPR) releases these ER-stress transducers from BiP. Released ATF6 translocates to the nucleus to augment XBP-1 gene expression. Parallel activation of IRE1 enables splicing of XBP-1 mRNA, which encodes a transcription factor that stimulates several stress inducible genes. Released PERK activates eIF2α, which then inhibits translation of cap-dependent proteins to further protect cells from UPR progression. Thus, cross-talks between these ER-stress transducers act via parallel pathways to facilitate cell survival and restore cellular homeostasis following a mild and transient ER-stress. However, under prolonged or severe ER-stress, the cap-independent translation of ATF4 continues. Nuclear ATF4 levels deregulate cellular homeostasis by enhancing the expression of ER-stress death sensors, CHOP and TRIB3. (B). TRIB3 acts as the ‘molecular switch’ that dictates cell survival or cell death decisions following ER-stress. Low levels of TRIB3 functions via a negative feedback-loop to suppress ATF4 and CHOP expression, thus enabling cell survival (left panel). However, high levels of TRIB3 down-regulates the AKT survival pathway, but does not suppress ATF4 and CHOP which continues to produce uncontrolled levels of TRIB3. This imbalance subverts the UPR and ER-stress responses from a survival mode towards apoptosis (right panel).

Studies provide clear evidence of cross-talks between multiple signal transduction pathways that regulate cell fate decisions following ER-stress induction in cancer cells [7], [15] (Please refer to Fig. 1A for a detailed description). A mild level of ER-stress activates a survival response called the Unfolded Protein Response (UPR). However, severe ER-stress subverts this UPR towards a pro-apoptotic pathway, which is dictated by the expression of ER-stress induced transcription factors ATF4 and CHOP, and the ER-stress induced death sensor TRIB3. Interestingly, under mild ER-stress, low TRIB3 levels act as a negative regulator of ATF4 and CHOP which favors cell survival. However, during severe ER-stress, high levels of ATF4 and CHOP augment TRIB3 expression and a parallel suppression of AKT, which favor apoptosis [16]–[18]. Therefore, TRIB3 seems to function as a master ‘molecular switch’ for survival *vs.* death signaling in cancer cells undergoing ER-stress (Fig. 1B). Thus, pharmacological agents that induce high TRIB3 levels should sensitize cancer cells to chemotherapy.

Thapsigargin (Tg), a well-known ER-stress inducer, can sensitize PC-3 cells to both paclitaxel and DTX [11]. In addition, the AKT inhibitor (AKTi-IV) can sensitize both HeLa and SKOV3 cell lines to cisplatin and etoposide [19]. However, these experimental compounds cannot be used in patients since they manifest significant *in vivo* toxicities [11], [19]. Furthermore, clinical approval of new agents that safety targets AKT and ER-stress would be an expensive and time-consuming process. In this respect, drug repositioning is becoming a very rewarding anti-cancer and chemosensitizing strategy [20]. We investigated whether two approved pharmacological agents, i.e. nelfinavir and curcumin, known to target the ER-stress and AKT pathways, can increase DTX’s anti-tumor efficacy against CRPC cells.

Nelfinavir (NFR) is one of the first HIV-1 protease inhibitors (HPI) to be clinically approved [21], [22] and is currently being repositioned as an anti-cancer agent, as well (ClinicalTrials.gov). Numerous studies have shown that NFR can both chemosensitize and radiosensitize a variety of different tumor cells [23]–[28]. Its sensitizing effects have been linked to the induction of ER-stress and the inhibition of AKT pathway [28]. Indeed, ritonavir, another HPI with similar mechanism of action, was also shown to enhance the anticancer effects of DTX in a highly aggressive PCa cell line, DU-145 [29]. However, the sensitizing effects of NFR are only exhibited at concentrations of ≥10 µM, which is higher than its safe and physiologically-achievable levels, i.e. 4.5–6 µM [30], [31]. Hence, the combination of NFR with another safe compound that similarly targets ER-stress and AKT pathways may be more efficacious.

Curcumin (CUR) is the active component of *Curcuma longa*, an East-Indian plant. This phytochemical has well known anti-inflammatory and anti-cancer properties and a number of laboratories are investigating it’s utility as an adjunct to chemotherapy [32]. Indeed, CUR has been shown inhibit the PI3K/AKT pathway and induce low levels of ER-stress specifically in cancer cells [33], [34]. In lung cancer cells, CUR exhibited synergistic anti-tumor effects when combined with DTX [35]. Recently, CUR was also shown to trigger cell death in colon cancer cells via ER-stress induced autophagy [36]. However, similar to NFR, the *in vitro* chemosensitizing effects of CUR were evident only at high concentrations (≥10 µM), which is difficult to achieve *in vivo* [37], [38]. Therefore, we hypothesized that CUR and NFR combination should potently increase their individual chemosensitizing abilities.

Investigations using C4-2B cells (a CRPC cell line) showed significantly increased anti-tumor efficacy of DTX following coexposure to NFR and CUR. Mechanistically, this 3-drug combination synergized to suppress AKT and induce ATF4, CHOP and TRIB3 levels. Both our *in vitro* and *in vivo* findings clearly implicate the potential of adjuvant therapy with physiologically achievable concentrations of NFR and CUR to augment the efficacy of DTX in CRPC patients.

## Materials and Methods

### Cell culture

The C4-2B cells, a bone metastatic CRPC subline derived from LNCaP, was a kind gift from Dr. Leland Chung’s laboratory (Emory University) [39]. These cells were grown in RPMI-1640 media (CellGro, Manassas, VA) supplemented with 10% fetal bovine serum (FBS) from Atlanta Biologicals (Lawrenceville, GA) and 1% penicillin/streptomycin antibiotic solution (CellGro). The RWPE-1 cells, a non-tumorigenic human prostatic epithelial cell line immortalized with human papillomavirus (HPV-18), were obtained from American type culture collection (ATCC; #CRL-11609). These cells were grown in keratinocyte serum free media (K-SFM) supplemented with epidermal growth factor (EGF) and bovine pituitary extract, all obtained from Invitrogen (Carlsbad, CA). Primary human prostate epithelial cells (PrEC) were obtained from ATCC (#PCS-440-010) and were cultured in prostate epithelial cell basal medium and supplements (ATCC; #PCS-440-030 and #PCS-440-040). The Bone Marrow Mesenchymal Stem Cells (BM-MSCs) were obtained from the stem cell core facility at Tulane University (New Orleans, LA) and were cultured in RPMI-1640 supplemented with 20% FBS and antibiotics. All cells were maintained at 37°C, in a humidified incubator containing 5% CO_2_.

### Reagents

Nelfinavir mesylate (NFR) powder was extracted and purified from 250 mg tablets (Agouron pharmaceuticals; San Diego, CA). Curcumin (CUR) was obtained from Acros Organics (Fair Lawn, NJ). Docetaxel (DTX) and Thapsigargin (Tg) were obtained from Sigma (St. Louis, MO) and the AKT-inhibitor (AKTi-IV; Catalog no. 124005) was obtained from Calbiochem (Billerica, MA). For *in vitro* studies, all drugs were dissolved in dimethyl sulfoxide (DMSO). Primary antibodies against total-AKT and phospho-AKT, total eIF2α and phospho-eIF2α, and against human BiP/Grp78, PARP and CHOP, were all purchased from Cell Signaling Technology (Danvers, MA). Antibodies against human TRIB3 and ATF4 were from Santa Cruz Biotechnology (Santa Cruz, CA), antibody against human β-Actin was from Fisher Scientific (Waltham, MA) and against Ki-67 was from Spring Biosciences (Pleasanton, CA). All secondary antibodies, such as goat anti-mouse, goat anti-rabbit and bovine anti-goat, were all purchased from Santa-Cruz Biotechnology.

### Cell viability assay

The MTT [3-(4,5-dimethylthiazol-2-yl)-2,5-diphenyltetrazolium bromide] assay was used to determine cell viability after exposure to the test compounds [40]. In brief, cells were plated in 96-well plates and allowed to adhere overnight. Desired concentrations of compounds, alone or in different combinations, were added to cells in 3 replicate wells. After 24–72 hr incubation, MTT (Sigma) was added to each well and incubated for 3 hr and formazan crystals were detected by purple coloration. Percent survival was calculated by measuring the absorbance at 540 nm using a µQuant plate reader from Bio-Tek (Seattle, WA).

### DNA fragmentation assay

The DNA fragmentation assay was carried out according to previous published studies [41]. Briefly, cells in 10 cm tissue-culture dishes were treated with the desired concentrations of DTX, NFR and CUR, alone and in combination. Cells were harvested after 24 hrs in a cell lysis buffer {0.2% Triton-X 100, 10 mM Tris-Cl (pH 7.4) and 10 mM EDTA (pH 8.0)}, followed by treatment with 100 µg/ml RNAse A (Sigma) and 0.5 mg/ml Proteinase-K (Sigma). The low molecular weight DNA were extracted by adding equal volumes of phenol, chloroform and isoamyl-alcohol (25∶24∶1) and additionally by chloroform and isoamylalcohol (24∶1). Extracted DNA was ethanol precipitated (300 mM NaCl and 100% ice-cold ethanol), redissolved in 1X TE (tris/EDTA) buffer and electrophoresed in a 2% agarose gel containing ethidium bromide (0.1 µg/ml). DNA fragmentation was visualized under UV-light using the Quantity One software (Bio-Rad; Hercules, CA).

### Caspase-3 assay

The EnzChek Caspase-3 assay Kit (Molecular Probes; Eugene, OR) detects apoptosis by measuring proteolytic cleavage of an amino-methylcoumarin (AMC) derived fluorescent substrate, Z-DEVD-AMC. Briefly, cells in 10 cm petri-dishes were treated with DTX, NFR and CUR, alone and in combination. Cells were harvested at 24 hrs, lysed, and Caspase-3 assay was carried out according to manufacturer’s protocols. Mean fluorescence intensities were measured by using an Flx800 microplate reader (BioTek) with excitation and emission wavelengths set at 360±20 nm and 460±20 nm, respectively.

### Western immunodetection

Whole cell lysates were harvested at different time points (30 mins to 9 hrs) post-exposure to DTX, NFR and CUR treatments by using 1X cell lysis buffer (Cell Signaling Technology; Danvers, MA). Proteins were quantified using the BCA protein assay reagent (Thermo Scientific; Rockford, IL). Approximately 30 µg of protein was fractionated onto 10% SDS-PAGE gels from Bio-Rad (Hercules, CA) and transferred to a PVDF membrane. Non-specific binding was blocked by incubating membranes with a bløk–CH chemiluminescent blocker (Millipore) and hybridized with desired primary antibodies (1∶1,000 dilution) overnight at +4°C and then with the HRP-labeled secondary antibodies (1∶5,000 dilution) for 1 hr at room temperature. Bands were detected using enhanced chemiluminescence (ECL) and the SuperSignal West Pico substrate (Thermo Scientific). Band intensities were quantified using the Image-J software (NIH) and densitometric value for each protein was normalized to the corresponding β-actin levels in each sample.

### Colony Forming Units assay

C4-2B cells were seeded in 6 cm dishes with 200 cells/dish. Drugs, alone or in combination, were added after 48 hrs and treatments were carried out in 3 replicate wells. Both NFR and CUR were replenished twice a week and DTX was replenished once a week along with fresh growth media. After three weeks, colonies were fixed with 100% ethanol and stained with methylene blue, and colony forming units (CFU) were enumerated by using the Quantity One software (Bio-Rad).

### Tumor xenograft studies

All experimental protocols involving laboratory animals were performed in accordance with NIH guidelines and were approved by the Institutional Animal Care and Use Committee at Tulane University (IACUC; Protocol #4295). The *in vivo* antitumor efficacy of DTX, alone or in combination with NFR and CUR, were determined in tumor xenografts in athymic nude mice (NCI; Frederick, MD). For each mouse (4-week old), C4-2B cells (2×10^6^) were resuspended in 100 µl of serum-free media and were injected subcutaneously (s.c.) along with 100 µl of Matrigel (BD Biosciences, San Jose, CA). When tumors reached a volume of 50–75 mm^3^ (∼2 weeks after injection), animals were randomized for treatment with either vehicle, DTX (10 mg/kg), or with DTX (10 mg/kg) + NFR (20 mg/kg) + CUR (100 mg/kg) by intraperitoneal (i.p.) injection. Before each injection, drugs were freshly dissolved in their respective vehicles [23], [43], [44]. DTX was administered once a week, and NFR and CUR were administered 5 days/week. Tumor sizes were measured twice a week by using a Vernier caliper and tumor volumes were calculated by using the formula, 0.5×length×width^2^ [45]. Weight of each mouse was measured and ratios of tumor-volume to total-weight were calculated at each time point. Tumors were excised at the end of the treatment period (4 wks), paraffin-embedded and sectioned for immunohistochemical (IHC) staining.

### Immunohistochemistry

Tumors were fixed in 10% neutral buffered formalin for 24 h followed by 70% ethanol and were then embedded in paraffin. Sections (∼5 µm) were cut and stained with hematoxylin and eosin (H&E). IHC for Ki-67 expression was carried out to determine the number of proliferating cells. Briefly, sections were deparaffinized, hydrated, and antigen retrieved by using 10 mM citrate buffer (pH 6.0). Sections were first blocked with 3% H_2_O_2_ and then with 1.5% blocking serum (Vectastain ABC kit, Vector laboratories; Burlingame, CA). Sections were then incubated with the anti-Ki-67 antibody for 30 min at room temperature. Following two washes in PBS, sections were incubated with biotinylated secondary antibody and then with the enzyme reagent (Vectastain ABC kit; Vector laboratories). Sections were stained with diaminobenzidine (DAB) and counterstained using Hematoxylin nuclear stain (Vector laboratories; #H-3401). Permanent mounting media was added and Ki-67 staining was visualized and captured by using an Eclipse E-400 microscope (Nikon Instruments, Melville, NY). In each slide, 5 different fields were visualized for Ki-67 stained cells and quantified by using the Image-J software.

### TUNEL assay

This DeadEnd Colorimetric TUNEL assay kit (Promega; Madison, WI) was used to determine apoptotic cells in tumor sections, according to the manufacturer’s protocols. This assay measures biotinylated nucleotide incorporation in DNA, which is then visualized by HRP-labeled streptavidin and DAB. Staining was visualized by Eclipse E-400 microscope and images were captured from 4 different fields in each tumor section.

### Synergy determination

The CalcuSyn software (Biosoft, Cambridge, UK) was used to calculate the combination index (CI) based on the Chou-Talalay method [46]. This method is based on the median-effect equation which includes Michaelis-Menton, Hill and Henderson-Hasselbalch equations and provides a quantitative measurement for additive (CI = 1), synergistic (CI<1) or antagonistic (CI>1) effects.

### Statistical analysis

Statistical analyses were carried out with the GraphPad Prism version-4.00 Software (San Diego, CA, USA). Results are expressed as standard error of means (±SEM). Significant changes from controls were determined by a two-tailed Student’s t-test and p-values of <0.05 were considered significant.

## Results

### Combined exposure to Thapsigargin and AKT-inhibitor sensitizes C4-2B cells to DTX-induced cytotoxicity

**Figure 2:**
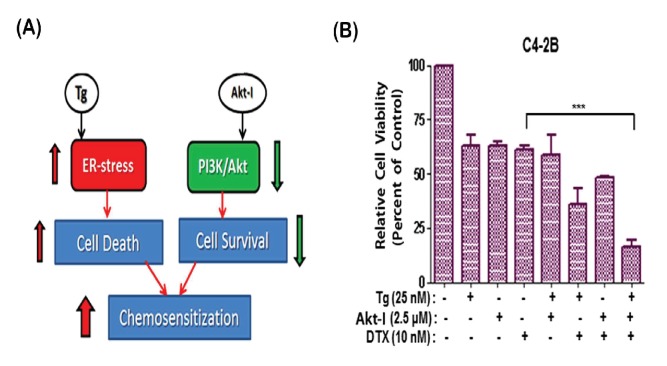
Chemosensitizing effects of simultaneous ER-stress induction and AKT suppression. (A). Simultaneous induction of ER-stress by Thapsigargin (Tg) and suppression of AKT by an Akt-inhibitor (Akt-I) may render cancer cells susceptible to the cytotoxic actions of chemotherapy. (B). Cytotoxic effects of DTX, alone or in combination with Tg and/or Akt-I, on C4-2B cell viability. Coexposure to Tg (25 nM) and Akt-I (2.5 µM) enhanced DTX (10 nM) induced cytotoxicity at 72 hrs post exposure (n = 3). Error bars represent ±SEM values and significant differences are shown as P-values (***, p<0.0005).

To address our central hypothesis that simultaneous targeting of ER-stress and AKT pathways will result in chemosensitization of CRPC cells, we first examined whether Tg and AKTi-IV coexposure can sensitize C4-2B cells to DTX-induced cytotoxicity (Fig. 2A). The effects of increasing drug concentrations and the time of exposures were first monitored by MTT-assays. At 72 hrs post exposure, the IC_50_ values for DTX, Tg and AKTi-IV were 35.8 nM, 80.8 nM and 5.5 µM, respectively (Table S1 in File S1). Possible synergistic effects of drug combination were then investigated using concentrations lower than their respective IC_50_ values. Coexposure studies clearly showed that the combination of DTX (10 nM), Tg (25 nM) and AKTi-IV (2.5 µM) resulted in a significant (p<0.0005) decrease in C4-2B cell survival, as compared to DTX alone (Fig. 2B). Future studies were carried out to investigate whether coexposure to two safe and approved agents, i.e. NFR and CUR, can similarly increase DTX sensitization of C4-2B cells.

### Coexposure to physiologically-achievable concentrations of NFR & CUR sensitizes C4-2B cells to DTX-induced cytotoxicity

**Figure 3:**
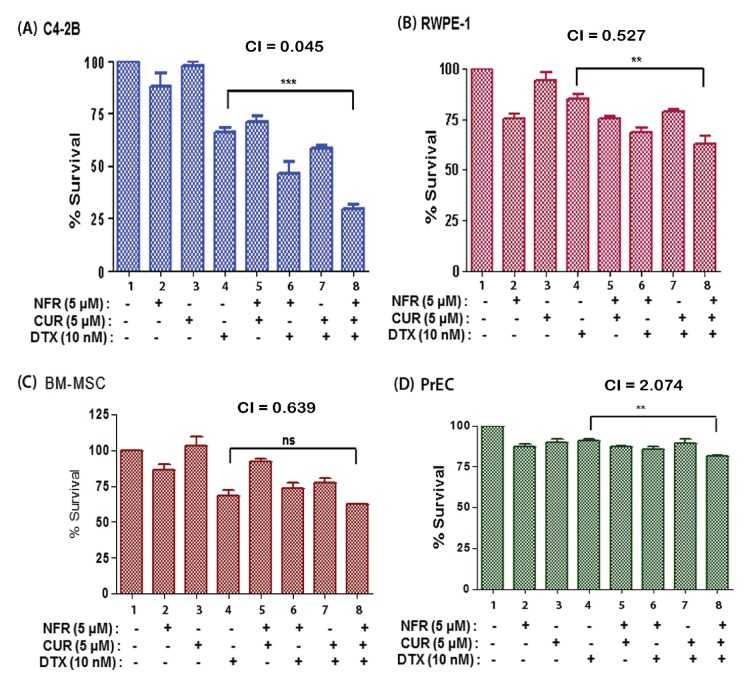
The DTX, NFR & CUR combination manifests synergistic cytotoxicity in C4-2B cells. Cytotoxic effects of DTX (10 nM), alone and in combination with NFR (5 µM) and/or CUR (5 µM), are shown in the following four cell types, (A) C4-2B, (B) RWPE-1, (C) PrEC and (D) BM-MSCs. Cell viability assays were carried out at 24 hrs post-exposure and percent change in cell survival, as compared to the untreated cells (control), were determined. The MTT-assays were carried out in 3 replicate wells and all experiments were repeated at least three independent times (n = 3). Error bars represent ±SEM and significant differences between DTX-only and the DTX+NFR+CUR group are shown as P-values (***, p<0.0005; **, p<0.005).

The IC_50_ values for DTX, NFR or CUR were calculated after exposure to individual drugs for 24, 48 and 72 hr (Table S1 in File S1). A concentration and time-dependent suppression in cell survival (MTT-assay) was observed. In subsequent studies, the sub-IC_50_ concentrations of drugs were used in 2- or 3- drug combinations and cell survival was monitored in different cell types, (A) C4-2B, (B) RWPE-1, (C) BM-MSC and (D) PrEC (Fig. 3). Although at 24 hrs the IC_50_ for DTX, NFR and CUR alone were shown to be 590.5 nM, 30.3 µM and 59 µM, respectively, a significant increase (p<0.0005) in C4-2B cytotoxicity was observed when DTX was used in combination with NFR and CUR. Interestingly, the rapid cytotoxicity observed in C4-2B cells was not evident in the non-tumorigenic RWPE-1 cells or in the primary cells, i.e. BM-MSCs and PrECs. The combination index (CI) analysis showed a value of 0.045 in C4-2B cells, suggesting a strong synergistic effect of the 3-drug combination. However, the CI values for RWPE-1 cells and BM-MSC were found to be much higher, i.e. 0.527 and 0.639, respectively. Furthermore, the CI value in PrEC cells was 2.074, suggesting an antagonistic effect. Similar studies in another aggressive PCa cell line, PC-3, also indicated significant (p<0.0005) increase in DTX sensitization following coexposure to both NFR and CUR (Table S1 in File S1 and Fig. S1A in File S1). Interestingly however, unlike in C4-2B cells, drug synergism in PC-3 cells was only observed at 72 hrs post-treatment. Taken together, these results clearly indicated that coexposure to NFR and CUR can rapidly and synergistically augment DTX-induced cytotoxicity in the CRPC line C4-2B, but not in the non-tumorigenic line, RWPE-1. Therefore, molecular mechanistic studies were carried out to delineate the differential actions of this 3-drug combination in both cell lines.

### Differential effects of NFR & CUR combination on DTX-induced apoptosis in C4-2B and RWPE-1 cells

**Figure 4:**
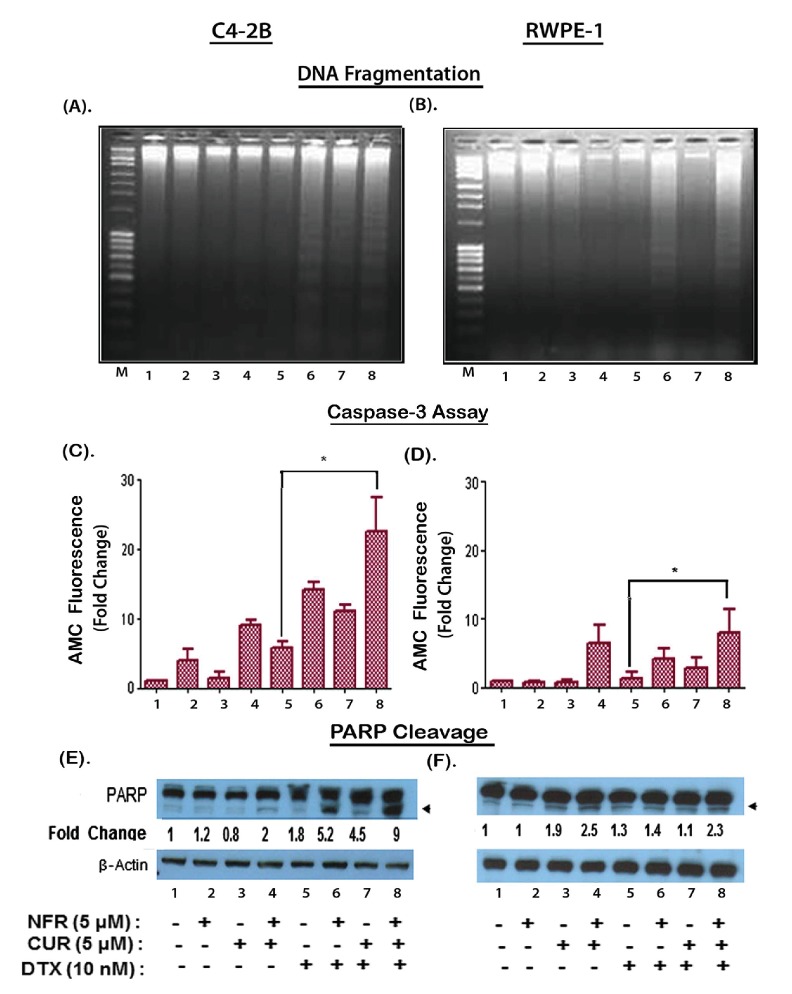
The NFR & CUR combination significantly increases DTX induced apoptosis in C4-2B cells. Induction of apoptosis in C4-2B (A, C, D) and RWPE-1 (B, E, F) cells following treatment with DTX (10 nM), alone and in combination with NFR (5 µM) and/or CUR (5 µM) was evaluated by DNA-fragmentation (A and B), Caspase-3 assay (C and E) and PARP cleavage (D and F). The DNA-fragmentation and Caspase-3 assays depict apoptosis after 24 hr post-exposure. Changes in PARP cleavage was measured at 9 hr post-exposure. The laddering pattern of fragmented DNA was indicative of apoptosis, which was further confirmed by increased AMC released by activated caspase-3 (n = 3) (*, p<0.05). Expression of both total and cleaved PARP was determined by western blot assays. The arrows indicate cleaved PARP levels. Fold changes in PARP cleavage in drug exposed cells (lanes 2–8) as compared to untreated cells (lane-1) are shown following normalization with respective β-actin levels.

We monitored whether the differential cytotoxicity in C4-2B *vs.* RWPE1 cells following treatment with the 3-drug combination is due to differences apoptosis (Fig. 4). Cellular apoptosis was evaluated by multiple techniques such as DNA fragmentation (Fig. 4A & 4B), caspase-3 induced AMC production (Fig. 4C & 4D) and PARP cleavage (Fig. 4E & 4F). The DNA fragmentation assay showed that the individual drugs did not induce apoptosis; however, an increase in laddering pattern was evident when DTX was combined with either NFR or CUR and apoptosis was further enhanced with the 3-drug combination. Although similar DNA fragmentation patterns were evident in RWPE-1 cells, the AMC assay clearly demonstrated a significantly higher Caspase-3 activity in C4-2B cells as compared to RWPE-1 cells. Neither NFR nor CUR alone was found to increase AMC production; however, their combined exposure increased Caspase-3 activity, and this was evident even in the absence of DTX cotreatment. Furthermore, although NFR or CUR alone could increase Caspase-3 activity in DTX exposed cells, the most significant increases were observed when both NFR and CUR were used in combination with DTX (compare lanes 5 and 8). Data from PARP cleavage assays further confirmed this differential effect of drug combination on apoptotic cell death. In C4-2B cells, a 9-fold increase was observed within 9-hrs post-exposure; however, only a 2.3 fold increase was seen in the RWPE-1 cells. Therefore, combined exposure to NFR and CUR profoundly increases DTX-induced apoptosis of the C4-2B cells.

### Combined treatment with DTX, NFR & CUR abrogates PI3K/AKT signaling in C4-2B cells

**Figure 5:**
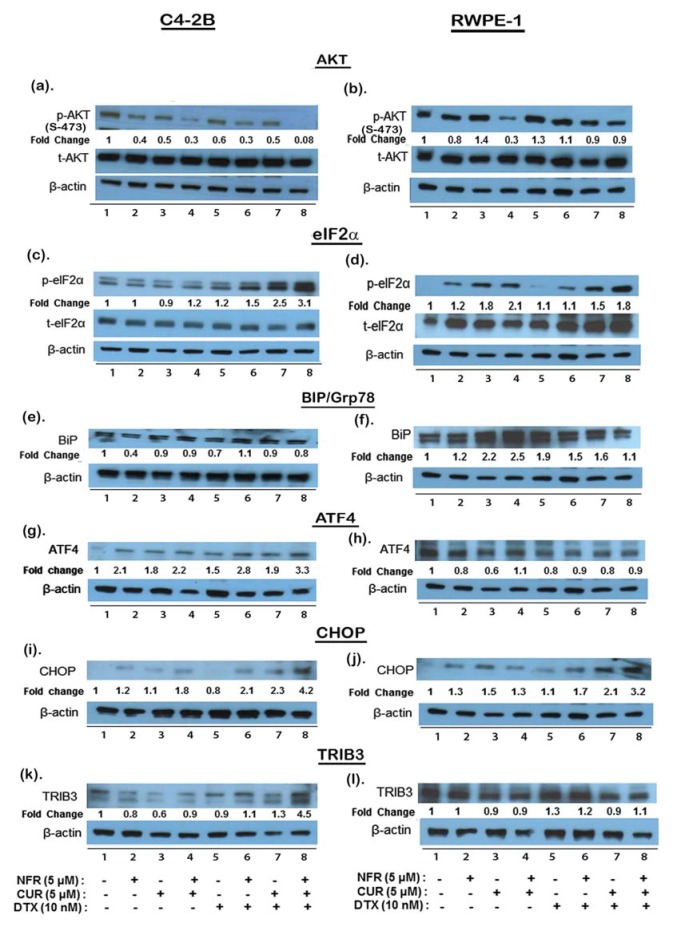
Differential effects of drug combination on PI3K/AKT signaling and activation of ER-stress transducers and death sensors. The effects of DTX (10 nM), NFR (5 µM) and CUR (5 µM), alone and in combination, on AKT-phosphorylation (a, b), ER-stress transducers (c–f), and ER-stress death sensors (g–l) were investigated by western blotting using lysates from both C4-2B and RWPE-1 cells. In panels (a) and (b), both total AKT (t-AKT) and IGF-1 induced AKT activation (p-AKT) levels are shown after 6 hrs post drug exposures. In panels (c–f), the expression of BiP and p-eIF2α, at 6 hrs of drug exposure is shown. The expression of CHOP and ATF4 following 3 hr drug treatment are shown in both C4-2B (g & i) and RWPE-1 (h & j) cells. Expression of TRIB3 following 6 hr drug treatment, alone or in combination, are shown in both C4-2B (k) and RWPE-1 (l) cells. Band intensities were quantified by the Image-J software. For both p-AKT and p-eIF2α quantifications, band intensities were first normalized to β-actin levels and then with either t-AKT or t-eIF2α levels, respectively. For all other proteins, normalization with β-actin levels was carried out. Treatment specific changes (lanes 2–8) are expressed as fold changes compared to untreated controls (lane-1).

To unravel the underlying mechanisms involved in chemosensitization by the 3-drug combination, western immunodetection studies were carried out to monitor both AKT activation and the expression of several ER-stress markers (Fig. 5 and Fig. S2 in File S1). Initial studies were carried out to monitor the time and dose dependent effects of DTX, alone or in combination with NFR and/or CUR, in the C4-2B cells (Fig. S2 in File S1). To determine their temporal effects on the AKT signaling pathway, C4-2B cells were first exposed to drugs from 30 min to 6 hrs and then stimulated with insulin-like growth factor-1 (IGF1; 10 ng/ml) for 15 min. Both total AKT (t-AKT) levels and AKT phosphorylated at serine-473 (p-AKT) were determined. As compared to unstimulated cells, p-AKT was increased by 3–4 fold following IGF1 stimulation (not shown). In C4-2B cells, the p-AKT suppression could be seen within 30 mins of exposure, and was clearly within within 3 hrs. Interestingly, although DTX alone was able to increase p-AKT levels within 30 min, this increase in AKT survival pathway was not seen in cells coexposed to NFR & CUR. The IGF-1 induced p-AKT levels were almost abolished at 6 hrs post-drug exposure (Fig. S2A in File S1). Neither the concentration nor the time of drug exposure was able to alter total AKT (t-AKT) levels in either cell type. In subsequent experiments (Fig. 5a & 5b), the optimum time and concentration of each agent was then used in combination regimen, and molecular effects on p-AKT were compared between the C4-2B (left) and RWPE-1 (right) cells. Most significant differences in p-AKT levels between the C4-2B and RWPE-1 cells were observed at the 6 hr time point. At this time point, although the drug combination could abolish p-AKT levels in C4-2B cells, only a slight decrease in p-AKT was evident in the RWPE-1 cells.

### Exposure to the 3-drug combination rapidly increases ER-stress transducers, BiP, eIF2µ and Xbp-1 in C4-2B cells

Studies were carried out to first compare the levels of BiP/Grp78 in both control (untreated) and drug exposed C4-2B and RWPE-1 cells (Fig. 5e & 5f). Constitutive basal expression of BiP (78 KDa) was indicative of inherent ER-stress in these two cell lines. However, no further increases in BiP were observed in C4-2B cells even at 9 hrs post-exposure to the drug combination (Fig. 5e and Fig. S2-C in File S1). Although 6 hr post-exposure to CUR alone and in combination with NFR could slightly increase BiP levels in the RWPE-1 cells (2 to 2.5 fold), this increase was not observed in cells exposed to the 3-drug combination. Interestingly however, both the total expression and the activation (phosphorylation) of another ER-stress transducer, eIF2µ (38 KDa) was clearly altered (Fig. 5c & 5d). Significant increases in p-eIF2µ were evident within 6 hrs post-exposure to the 3-drug combination. Interestingly however, in contrast to the C4-2B cells, the RWPE-1 cells showed lower basal p-eIF2µ which increased significantly following drug exposures. Furthermore, although total eIF2µ (t-eIF2µ) was not affected in C4-2B cells, it was detectably increased in the RWPE-1 cells. This differential induction of p-eIF2µ was further confirmed by calculating the p-eIF2µ to t-eIF2µ ratios, which were 3.1 fold in C4-2B cells and only 1.8 fold in RWPE-1 cells. Thus, a more robust activation of eIF2µ was seen in C4-2B cells, which occurred independent of changes in their BiP levels. Therefore, we wanted to further confirm ER-stress induction by monitoring another downstream ER-stress transduced factor, XBP-1 (Fig. S1B in File S1). Spliced XBP-1 (sXBP-1) mRNA levels has been shown to be a highly sensitive marker of ER-stress in NFR exposed cells [47] and can be easily quantified by qRT-PCR [42]. In C4-2B cells, we observed a very rapid increase in the sXBP-1 mRNA levels within 3 hrs of exposure to the 3-drug combination. This substantial increase was not seen in cells exposed to DTX alone or in cells exposed to the individual drugs (Fig. S1B in File S1). Therefore, our findings clearly indicated a differential molecular effect of the 3-drug combination in inducing ER-stress in the C4-2B cells, but not in the RWPE-1 cells. This differential effect may be directly associated with the enhanced apoptosis observed in the aggressively growing CRPC cells.

### The 3-drug combination rapidly enhances ER-stress associated death sensors, CHOP, ATF-4 and TRIB3 in C4-2B cells

Although the activated eIF2α (p-eIF2α) can facilitate cell survival by inhibiting the synthesis of cap-dependent proteins, an UPR response, the expression of ATF4, a cap-independent transcription factor, continues. ATF4 in turn regulates the expression of several downstream ER-stress death-sensors, i.e. CHOP and TRIB3 [16]. A negative feedback by TRIB3 can also regulate ATF4 and CHOP expression and inhibit AKT phosphorylation [18] (Please see Fig. 1). We documented differential effects of the 3-drug combination on ATF4 (38 KDa), CHOP (27 KDa) and TRIB3 (45 KDa) expression in C4-2B and RWPE-1 cells (Fig. 5g–l). In C4-2B cells, ATF4 expression was increased (3.3 fold) within 3 hr post-exposure to the drug combination (Fig. 4g). Interestingly however, ATF4 expression was slightly decreased in the RWPE-1 cells (Fig. 4h). Although CHOP expression increased in both C4-2B (4.2 fold) and RWPE-1 (3.2 fold) cells within 3 hr post exposure (Fig. 4i & 4j), significantly increased TRIB3 expression (4.5 fold) was only observed in the C4-2B cells (Fig. 4k) but not in the RWPE-1 cells (Fig. 4l). These findings suggested that the CHOP/ATF4/TRIB3 signaling axis is significantly enhanced following exposure to the drug combination and results in the enhanced cell death. Interestingly, this increase in CHOP/ATF4/TRIB3 axis progresses more rapidly in the C4-2B cells and is facilitated by coexposure to the NFR & CUR combination.

### Suppressive effect of DTX on C4-2B cell clonogenic ability is further exacerbated by coexposure to NFR & CUR combination

**Figure 6:**
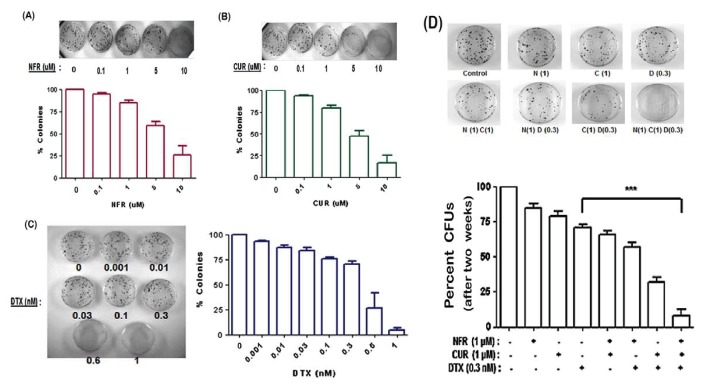
Long-term effects of our three-drug combination on the clonogenic ability of C4-2B cells. Cells were exposed to increasing concentrations of individual agents, (A) NFR (0.1–10 µM), (B) CUR (0.1–10 µM), or (C) DTX (0.001–1.0 nM) for 3 wks. As compared to controls, percent change in clonogenic abilities in drug exposed groups were determined by CFU assays. (D) Subtoxic concentrations of each drug, as determined from A–C, was used in a combination consisting of DTX (0.3 nM), NFR (1 µM) and CUR (1 µM). Percent change in CFUs were enumerated. Representative images of stained colonies are shown above each treatment panel. Bar graphs represent data generated from three independent experiments (n = 3) carried out using triplicate 60 mm dishes. Error bars represent ± SEM. In (D), significant differences between DTX-only and the DTX+NFR+CUR group is shown as ***, p<0.0005.

*In vitro* studies were carried out to examine the chronic (long-term) effects of drug combination on the clonogenic potential of C4-2B cells (Fig. 6). Cells (200 cells/dish) cultured for 3 weeks in the absence or presence of drugs, alone or in combination, showed significant differences in the number of colony forming units (CFU). Initially, individual compounds were tested over a range of concentrations (Fig. 6, A–C). Sub-toxic concentrations of DTX (0.3 nM), NFR (1 µM) and CUR (1 µM) were then used in the combination studies (Fig. 6D). As compared to their acute effects on cytotoxicity, chronic exposure to significantly lower concentrations of individual agents showed a more pronounced suppressive effect on CFUs. Importantly, coexposure to DTX+NFR+CUR combination showed the most drastic (p<0.0005) reduction in CFUs (92%), as compared to DTX alone (34%). These *in vitro* findings using CFUs suggested that chronic exposure to our 3-drug combination may also depict significant anti-tumor effects *in vivo*.

### NFR & CUR combination enhances the anti-tumor efficacy of DTX in C4-2B tumor xenografts

**Figure 7:**
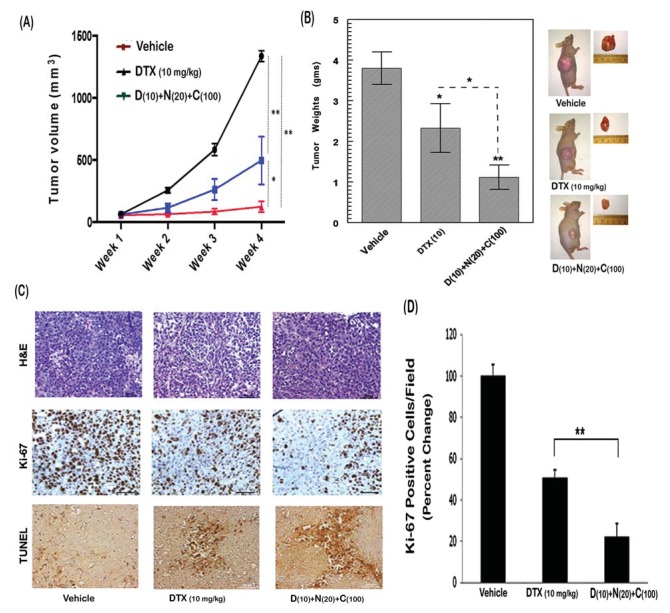
Chronic anti-tumor effects of DTX, NFR & CUR combination on C4-2B tumor xenografts. C4-2B cells (2×10^6^) were injected subcutaneously (s.c.) in mice and drug treatments were initiated when tumors reached a volume of 50–75 mm^3^. (A) Results show average tumor volumes in either vehicle treated group or after 1–4 wks of exposure to DTX (10 mg/kg/1x per wk) alone or following combined exposure to DTX (10 mg/kg/1x per wk), NFR (20 mg/kg/5x per wk) and CUR (100 mg/kg/5x per wk). Average tumor volumes from three to five mice are shown in the line graphs. (B) Average tumor weights in untreated mice and following 4-wks of drug treatment are shown in the bar graphs. A representative image of the tumor-bearing mice and the excised tumors are also shown. (C) A representative image of IHC-stained tumor sections from control (vehicle) and drug treated groups are shown. Panels depict H&E staining (top), Ki-67 staining (middle) and TUNEL staining (bottom). (D) Quantitative analysis of Ki-67 stained cells in tumor sections (five independent fields) from control (vehicle) and drug treated groups. Error bars represent ±SEM. Significant differences between DTX-only and the 3-drug combination group, *, p<0.05 and **, p<0.01.

The anti-tumor effects of chronic exposure to DTX (10 mg/kg), alone or in combination with NFR (20 mg/kg) and CUR (100 mg/kg) were monitored in athymic nude mice transplanted subcutaneously (s.c.) with C4-2B tumor xenografts (Fig. 7). Drug treatments were initiated at two-weeks after C4-2B cell injection when tumors reached a volume of 50–75 mm^3^ and tumor volumes were measured weekly for up to 4 wks post drug initiation. In control (untreated) mice, a 22-fold increase in tumor-volume was seen after 4 wks. However, in both the DTX-only group and the DTX+NFR+CUR group, C4-2B tumor growth was significantly attenuated. In DTX only group, the relative increase in tumor volume was 7-fold and only a 2-fold increase was documented in the 3-drug group (Fig. 7A). Tumors were excised at 4-weeks and tumor weights (grams) were determined (Fig. 7B). A significant (p<0.05) increase in DTX-induced anti-tumor efficacy was evident following adjunct therapy with NFR and CUR. The IHC analysis of tumor sections showed significant differences in the rates of cell proliferation (Ki-67) and cell death (TUNEL staining) (Fig. 7C). Quantitative analysis of Ki-67 stained cells showed ∼55% decrease with DTX alone and a more than 80% decrease with the 3-drug combination (Fig. 7D). TUNEL staining also showed significant increases in apoptotic cells as compared to the control (untreated) group. These observations clearly indicated that physiologic doses of NFR and CUR can increase the anti-tumor efficacy of DTX against CRPC tumors *in vivo*.

## Discussion

There is an urgent need to increase the susceptibility of CRPC cells to DTX therapy. Although at the current doses of DTX used a peak plasma concentration of ∼20 nM can be achieved within 24 hr post infusion [11], our *in vitro* studies indicated that the 50% cytotoxicity (IC_50_) at 24 hrs is significantly higher (Table S1 in File S1). Significant cell death with the physiologically-achievable concentration of DTX was only observed after 72 hrs. Therefore, in addition to increased DTX sensitivity, strategies to enhance the rate of DTX-mediated cell killing would be needed. Our *in vitro* investigations clearly showed that a rapid and substantial chemosensitizaton of CRPC cells can be accomplished with a drug combination that simultaneously targets ER-stress and AKT pathways. The utility of combining an approved drug (NFR) and a safe phytochemical (CUR) may be rapidly implemented in patients with CRPCs.

Although a number of independent publications have previously shown that NFR or CUR alone can chemosensitize cancer cells [27], [28], [35], [36], none of these studies demonstrated their combined efficacy. Importantly, we observed synergistic increases in CRPC chemosensitization at physiologically-achievable concentrations of these two agents, which further corroborated that dual targeting of ER-stress and AKT using these two chronically used drugs may be a very effective anti-tumor strategy. Although initial findings with the thapsigargin and AKT-inhibitor combination provided evidence of increased DTX cytotoxicity at 72 hrs post-treatment, the chemosensitizing ability of NFR and CUR combination was perceived within 24 hrs. Furthermore, this rapid cytotoxicity of our 3-drug combination, i.e. DTX+NFR+CUR, was more pronounced in the C4-2B cells, as compared to the RWPE1 cells. Most importantly, the primary cells, i.e. PrEC and BM-MSC, showed minimal toxicity with this drug combination, which further justified the advantage and safety of our therapeutic approach.

The high rate of growth in aggressive cancer cells may enable to be more susceptible to our ER-stress inducing combination; however, the differential effects observed in RWPE-1 and C4-2B cells, which have comparable growth rates, clearly indicated that the observed differences in apoptosis rates may be dependent upon an uncontrolled ER-stress propagation and the induction of ER-stress death sensors. Indeed, although both RWPE-1 and C4-2B cell lines showed constitutive basal levels of BIP/Grp78, the differential effects of our drug-combination in both suppression of activated (phosphorylated) AKT and in the induction of activated (phosphorylated) eIF2µ, clearly suggested different mechanisms of action. Basal levels of eIF2µ was much higher in C4-2B cells than in RWPE-1 cells which also implicated a higher metabolism in C4-2B cells that may be specifically targeted to augment the ER-stress induced apoptosis rather than survival.

The PI3K/AKT pathway is a crucial survival mechanism in cancer cells and its inhibition can activate mitochondrial pro-apoptotic pathways. Indeed, previous studies have shown that DTX exposure stimulates the PI3K/AKT signaling in PCa cells and promotes prostate tumor survival [7]. Recent findings also indicated that ER-stress modulates AKT substrate specificity and reduces both total and phosphorylated AKT [48]. Interestingly, AKT phosphorylation of Thr-308 was suppressed while that of Ser-473 was increased under ER-stress. These findings may provide a mechanistic explanation for the significant contrasting effects of modulating ER-stress and AKT signaling, observed between the transformed C4-2B cells and the normal PrEC cells (Fig. 3). Although studies using supra-physiological concentrations of DTX (75 nM) have also documented decreased p-AKT levels [48], our current investigations using physiologic concentrations of DTX (10 nM) clearly suggested that simultaneous targeting of both ER-stress and AKT pathways can disable the early induction of survival signaling in cancer cells [49].

Our findings support an earlier study showing that UPR is differentially activated depending on the cell type and the stressors being utilized [50]. Although high concentrations of DTX (20 nM) can induce ER-stress in a melanoma cell line [51], we did not document any significant increases in ER-stress following exposure to a lower DTX concentration (10 nM). Interestingly, even at this sub-toxic concentration of DTX, concomitant exposure to NFR and CUR resulted in the increased expression of both CHOP and ATF4. Thus, our findings suggest that the elimination of aggressive tumor cells, but not normal cells, may be possible by dysregulating the critical control of ER-stress in aggressive cancer cells.

Augmented CHOP and ATF4 levels have been associated with the increased expression of TRIB3 [18] and increased TRIB3 expression is dependent on the PI3K/AKT activation status [52]. In a recent report by Han *et al*, (2013), forced expression of ATF4 and CHOP was shown to increase oxidative stress by depleting ATP, which resulted in enhanced cell death [53]. In our study, exposure to the triple drug combination was associated with the highest increases in TRIB3 expression. Additionally, we have also shown an increase in caspase-3 activity and apoptosis in cells exposed to the triple drug combination, as compared to the individuals agents.

Although the exact mechanism/s associated with TRIB3-mediated regulation of cell survival and apoptosis is unknown, studies showed that TRIB3 is cleaved by caspases in cells that are undergoing severe stress [54]. During the early phases of ER-stress, where cell survival is promoted, TRIB3 prevents caspase-3 activation by nuclear translocation of pro-caspase 3. However, under prolonged or severe ER-stress, TRIB3 was shown to undergo cleavage by caspases and thus trigger further caspase activation and induction of apoptosis [17]. Although we did not measure cleaved TRIB3 levels, the persistent increases in TRIB3 expression and caspase-3 activation, clearly suggested this as a possible underlying mechanism of increased apoptosis in cells exposed to the 3-drug combination. Although TRIB3 expression was significantly enhanced in C4-2B cells, where the inherent ER-stress, i.e. eIF2µ was found to be much higher, increased TRIB3 expression was not evident in RWPE-1 cells.

Interestingly, although CUR or NFR exposures alone were able to increase ATF4 and CHOP expression, TRIB3 levels were downregulated (Fig. 5). We postulated that this happens in order to ensure cell survival following low levels of ER-stress. Although TRIB3 expression is increased by both ATF4 and CHOP [18], at low levels of ER-stress TRIB3 initially downregulates its own expression by inhibiting ATF4/CHOP expression. However, this negative feedback via TRIB3 is not functional during prolonged and severe ER-stress, where continuous increases in TRIB3 is not regulated by the ATF4/CHOP axis, and suppression of Akt [50] and activation of caspases [17], [54] may ultimately switch cells towards a pro-apoptotic cascade. Indeed, although NFR or CUR alone could induce ATF4 (∼2.1 and ∼1.8 fold), no parallel increases in CHOP expression was seen at 3 hrs post-exposure (Fig. 5). This suggested that in addition to the suppression of AKT, suppression of both ATF4 and CHOP may be needed for the substantial increases in TRIB3 expression observed in our studies on C4-2B cells.

Results obtained with the acute cytotoxicity of the 3-drug combination (Fig. 2) were corroborated by our chronic exposure studies *in vitro* (Fig. 6) and *in vivo* (Fig. 7). In C4-2B tumor xenografts, chronic exposure to NFR (20 mg/kg/5x per wk) & CUR (100 mg/kg/5x per wk) combination significantly enhanced the anti-tumor efficacy of DTX (10 mg/kg/1x per wk). As compared to a 22-fold increase in untreated mice, DTX alone showed 7-fold increase and the 3-drug treated group showed only a 2-fold increase in tumor growth. No significant anti-tumor effects were seen in mice treated with NFR or CUR alone or with NFR & CUR combination (data not shown). Since our primary focus was to document the chemosensitizing effects of NFR and CUR combination, we did not carry out studies using the DTX and NFR or the DTX and CUR treatment groups. Previous publications in lung [24], prostate [25] and breast cancer [27] models had used much higher concentrations of NFR (>60 mg/kg/day) to document anti-tumor effects. However, our findings showed that much lower concentration of NFR (20 mg/kg/5-days a week) was needed when combined with CUR. This enables a safer dose of NFR to be used for the sensitization of cancer cells.

Interestingly, the rapid (24 hrs) cytotoxic effects observed in C4-2B cells were not evident in PC-3 cells. Increase in DTX’s cytotoxic effects following NFR and CUR coexposure were observed only at 72 hrs (Fig. S1A in File S1). The androgen receptor (AR) negative PC-3 cells are known to be more resistant to DTX [55]. Since p53 status is a crucial determinant of DTX sensitivity [56], the p53 null PC-3 cells may be inherently resistant to DTX. However, despite the delayed response, efficacy at which our 3-drug combination was able to potentiate DTX-induced cytotoxicity in the PC-3 cells clearly indicated the therapeutic potential of this regimen against these highly aggressive AR-negative CRPC cells, as well.

Although we have not investigated whether the NFR and CUR combination can increase the efficacy of other chemotherapeutic agents and whether this combination will be able to sensitize other cancer lines, our molecular mechanistic studies implicate that aggressive cancer cells may become more susceptible to cytotoxic chemotherapy when simultaneous suppression of AKT and induction of ER-stress is initiated as an adjunct regimen. In summary, NFR and CUR combination increases DTX-induced cell killing via a two-pronged approach, which led to CHOP/ATF4 mediated increase in TRIB3 and the switching of ER-stress from a survival mode towards an apoptotic mode (Fig. S3 in File S1). Our novel preclinical findings with NFR and CUR provide the proof-of-concept that this pro-apoptotic response of ER-stress may be exploited to specifically sensitize the CRPC cells to chemotherapy.

## Supporting Information

File S1 **Table S1**, *IC_50_ values of individuals drugs on C4-2B and PC-3 cell survival.* **Figure S1**, (A). *Cytotoxic effects of drug combination in PC-3 cells.* Percent change in cell viability following 72 hr exposures to DTX (10 nM) alone or in combination with NFR (5 µM) and/or CUR (5 µM) (n = 3; ***, p<0.0005). (B) *Effect of drug combination on XBP-1 mRNA in C4-2B cells.* Spliced XBP-1 mRNA expression in C4-2B cells was determined by qRT-PCR analysis. Cells were exposed to drugs for 30 min or 3 hrs, total RNA isolated, reverse-transcribed and PCR amplified. Fold changes (ΔCt) in XBP1 mRNA were calculated after normalization to GAPDH mRNA levels (n = 3). **Figure S2**, *Temporal effects of drug combinations on AKT and ER stress in C4-2B cells.* Immunoblots show the effects of drug exposure for 30 min, 3 hrs and 6 hrs. Temporal effects on (A). IGF-1 induced p-AKT and t-AKT; (B) p-eIF2α and t- eIF2α; (C) BiP/Grp78; (D) CHOP; (E) ATF4 and (F) TRIB3 levels are shown. Band intensities were normalized to β-actin levels. Treatment specific changes (lanes 2–8) are expressed as compared to controls (lane-1). **Figure S3**, *Proposed mechanism for the antitumor efficacy of triple-drug combination.* Simultaneous exposure to the DTX, NFR and CUR drug combination induces severe ER-stress, resulting in the up-regulation of CHOP, ATF4 and TRIB3. The augmented TRIB3 level suppresses the AKT survival pathway and further enhances ER-stress induced apoptosis by TRIB-3 induced caspase-3 activation. Therefore, coexposure to physiological concentrations of NFR & CUR can increase the susceptibility of CRPC cells to DTX therapy. **Methods S1**, *(1). PC-3 Cell culturing; (2). qRT-PCR analysis of XBP-1.*
(PDF)
