# Supplementary material for: Subverting ER-Stress towards Apoptosis by Nelfinavir and Curcumin Coexposure Augments Docetaxel Efficacy in Castration Resistant Prostate Cancer Cells
Source: PLoS One. 2014 Aug 14;9(8):e103109. doi: 10.1371/journal.pone.0103109 (PMC4133210; doi:10.1371/journal.pone.0103109)
Supplement: File S1 — Table S1, IC50 values of individuals drugs on C4-2B and PC-3 cell survival. Figure S1, (A). Cytotoxic effects of drug combination in PC-3 cells. Percent change in cell viability following 72 hr exposures to DTX (10 nM) alone or in combination with NFR (5 µM) and/or CUR (5 µM) (n = 3; ***, p<0.0005). (B) Effect of drug combination on XBP-1 mRNA in C4-2B cells. Spliced XBP-1 mRNA expression in C4-2B cells was determined by qRT-PCR analysis. Cells were exposed to drugs for 30 min or 3 hrs, total RNA isolated, reverse-transcribed and PCR amplified. Fold changes (ΔCt) in XBP1 mRNA were calculated after normalization to GAPDH mRNA levels (n = 3). Figure S2, Temporal effects of drug combinations on AKT and ER stress in C4-2B cells. Immunoblots show the effects of drug exposure for 30 min, 3 hrs and 6 hrs. Temporal effects on (A). IGF-1 induced p-AKT and t-AKT; (B) p-eIF2α and t- eIF2α; (C) BiP/Grp78; (D) CHOP; (E) ATF4 and (F) TRIB3 levels are shown. Band intensities were normalized to β-actin levels. Treatment specific changes (lanes 2–8) are expressed as compared to controls (lane-1). Figure S3, Proposed mechanism for the antitumor efficacy of triple-drug combination. Simultaneous exposure to the DTX, NFR and CUR drug combination induces severe ER-stress, resulting in the up-regulation of CHOP, ATF4 and TRIB3. The augmented TRIB3 level suppresses the AKT survival pathway and further enhances ER-stress induced apoptosis by TRIB-3 induced caspase-3 activation. Therefore, coexposure to physiological concentrations of NFR & CUR can increase the susceptibility of CRPC cells to DTX therapy. Methods S1, (1). PC-3 Cell culturing; (2). qRT-PCR analysis of XBP-1. (PDF) [file pone.0103109.s001.pdf]

**File S1.** **Contains Table-S1, Figures S1 to S3, and Methods S1**

Table 1. Effect of individual drugs on C4-2B and PC-3 cell survival.

| Hrs | C4-2B ($IC_{50}$) | | | | |
|---|---|---|---|---|---|
| | DTX (nM) | Tg (nM) | Akt inhibitor (μM) | NFR (μM) | CUR (μM) |
| 24 | 590.5 | 389.3 | 1273 | 30.3 | 59 |
| 48 | 167.4 | 345.6 | 20.61 | 11.8 | 23.8 |
| 72 | 35.8 | 80.8 | 5.5 | 6.7 | 6.4 |
| **Hrs** | **PC-3 ($IC_{50}$)** | | | | |
| | DTX (nM) | Tg (nM) | Akt inhibitor (μM) | NFR (μM) | CUR (μM) |
| 24 | 6429 | ND | ND | 26.4 | 61.4 |
| 48 | 1187 | ND | ND | 17.8 | 22.1 |
| 72 | 79.6 | ND | ND | 10.8 | 13.6 |

Note: The 50% inhibitory concentration ($IC_{50}$) values were determined by MTT-cell survival assays in three independent experiments (n=3) carried out in triplicate samples.

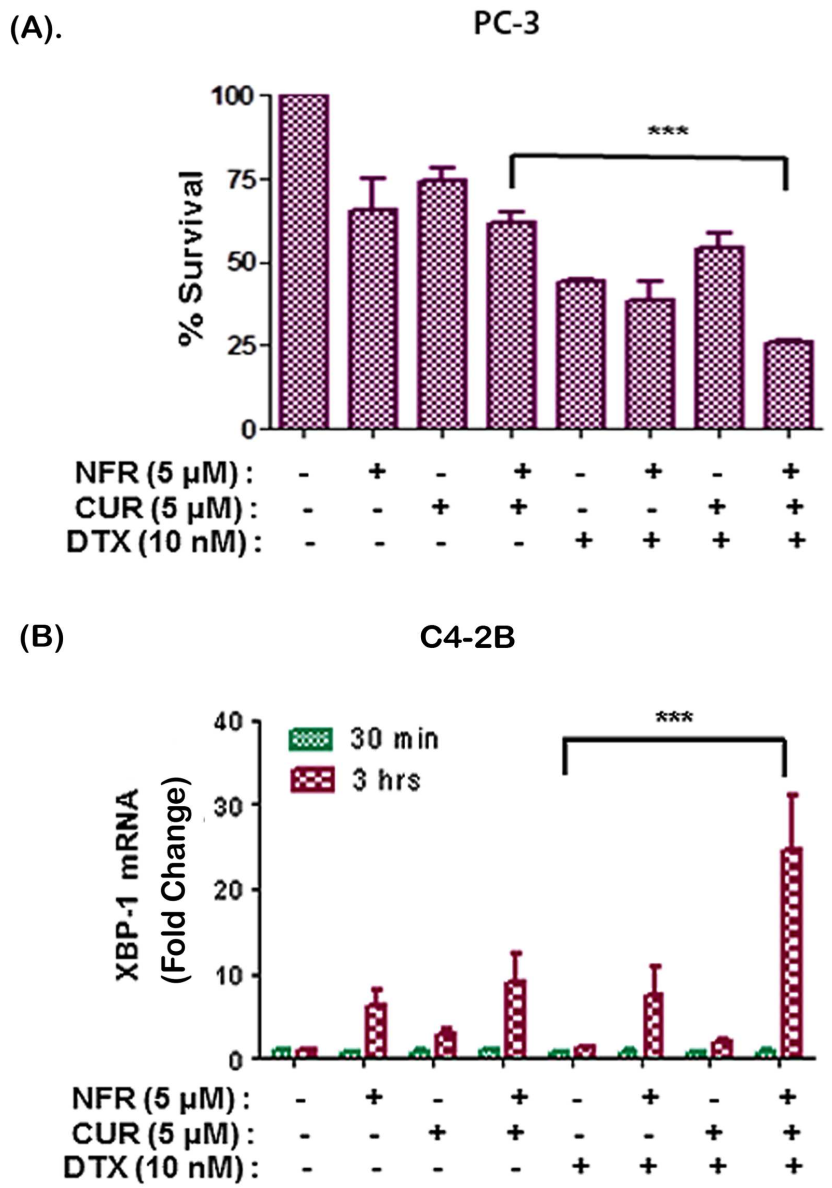
(A).
PC-3
% Survival
100
75
50
25
0
***
NFR (5 µM) : - + - + - + - +
CUR (5 µM) : - - + + - - + +
DTX (10 nM) : - - - - + + + +
(B)
C4-2B
XBP-1 mRNA (Fold Change)
40
30
20
10
0
30 min
3 hrs
***
NFR (5 µM) : - + - + - + - +
CUR (5 µM) : - - + + - - + +
DTX (10 nM) : - - - - + + + +

**Fig.S1**

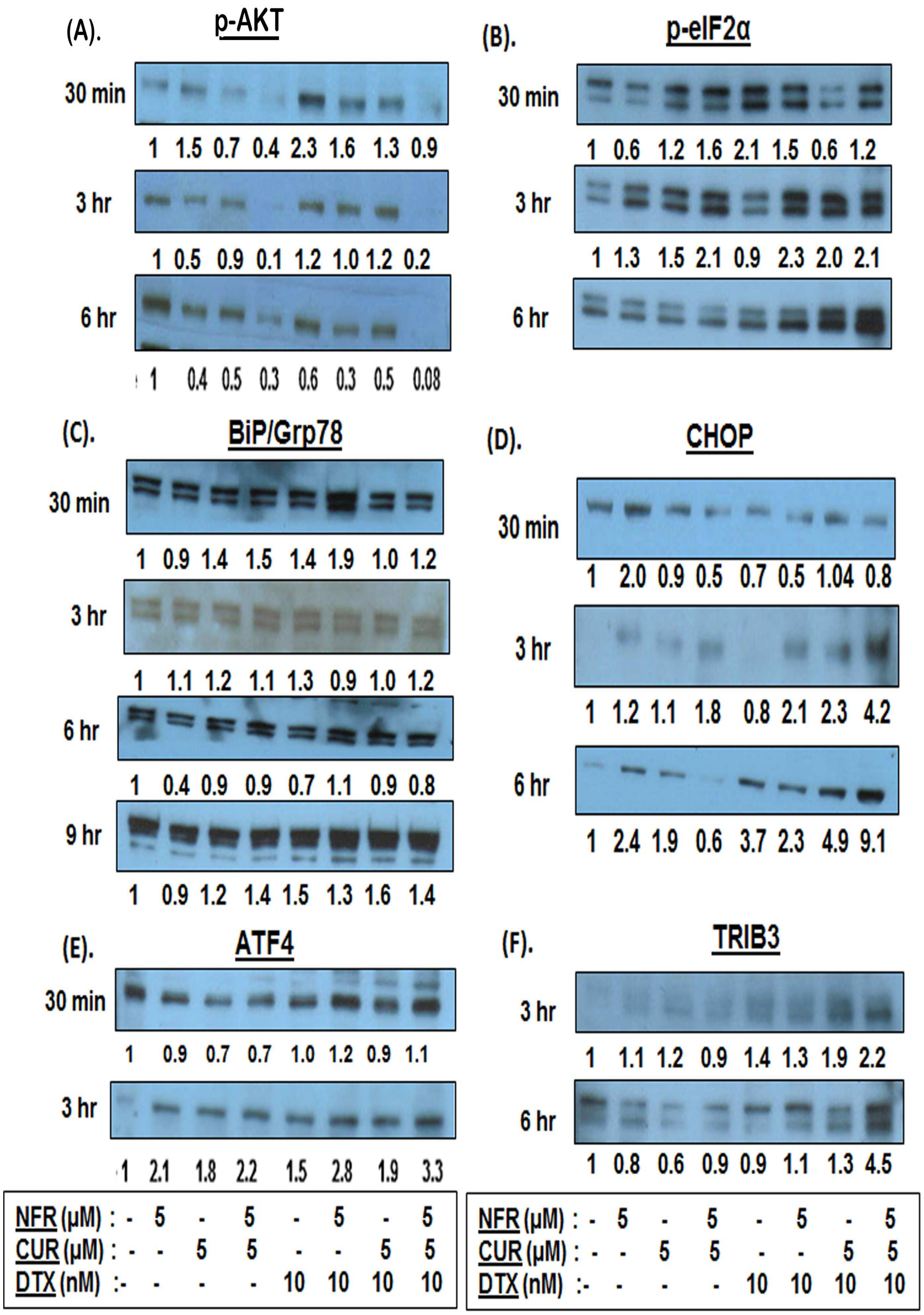
(A).
p-AKT
30 min
1 1.5 0.7 0.4 2.3 1.6 1.3 0.9
3 hr
1 0.5 0.9 0.1 1.2 1.0 1.2 0.2
6 hr
1 0.4 0.5 0.3 0.6 0.3 0.5 0.08
(B).
p-eIF2α
30 min
1 0.6 1.2 1.6 2.1 1.5 0.6 1.2
3 hr
1 1.3 1.5 2.1 0.9 2.3 2.0 2.1
6 hr
(C).
BiP/Grp78
30 min
1 0.9 1.4 1.5 1.4 1.9 1.0 1.2
3 hr
1 1.1 1.2 1.1 1.3 0.9 1.0 1.2
6 hr
1 0.4 0.9 0.9 0.7 1.1 0.9 0.8
9 hr
1 0.9 1.2 1.4 1.5 1.3 1.6 1.4
(D).
CHOP
30 min
1 2.0 0.9 0.5 0.7 0.5 1.04 0.8
3 hr
1 1.2 1.1 1.8 0.8 2.1 2.3 4.2
6 hr
1 2.4 1.9 0.6 3.7 2.3 4.9 9.1
(E).
ATF4
30 min
1 0.9 0.7 0.7 1.0 1.2 0.9 1.1
3 hr
1 2.1 1.8 2.2 1.5 2.8 1.9 3.3
(F).
TRIB3
3 hr
1 1.1 1.2 0.9 1.4 1.3 1.9 2.2
6 hr
1 0.8 0.6 0.9 0.9 1.1 1.3 4.5
NFR (μM) : - 5 - 5 - 5 - 5
CUR (μM) : - - 5 5 - - 5 5
DTX (nM) :- - - - 10 10 10 10
NFR (μM) : - 5 - 5 - 5 - 5
CUR (μM) : - - 5 5 - - 5 5
DTX (nM) :- - - - 10 10 10 10

**Fig. S2**

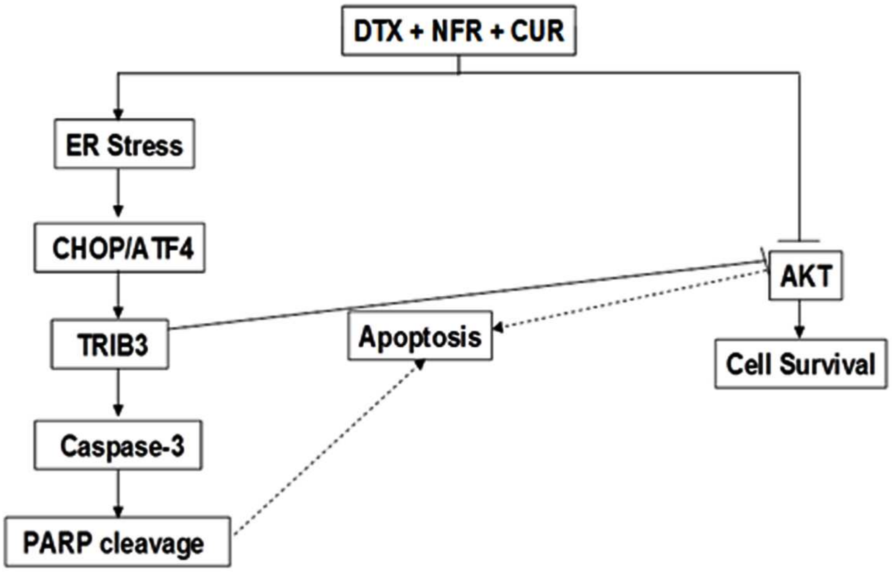
DTX + NFR + CUR
ER Stress
CHOP/ATF4
TRIB3
Caspase-3
PARP cleavage
Apoptosis
AKT
Cell Survival

**Fig. S3**

## Methods S1

**PC-3 Cell Culture.** PC-3 cells were obtained from ATCC (Manassas, VA; #CRL-1435). Cells were maintained in RPMI-1640 medium supplemented with 10% FBS (Atlanta Biologicals, Lawrenceville, GA), and antibiotics (100 U/ml penicillin and 100 µg/ml streptomycin). Cells were grown at 37℃, with 5% $CO_2$ and 95% humidified air.

**Quantitative Reverse-transcriptase Polymerase Chain Reaction (qRT-PCR).** Changes in spliced XBP-1 mRNA were measured by qRT-PCR, according to Hirota et al, (2006) [41]. Total RNA was isolated by using the Trizol$^{TM}$ reagent (Invitrogen). The cDNA were synthesized using iScript reverse transcription (RT) supermix (Bio-Rad) and oligo-dT as primer, and using the following protocol: priming for 5 min at 25ºC, reverse transcription for 30 min at 42ºC, and inactivation for 5 min at 85ºC. The PCR primer sequences used for XBP-1 were: <sense> 5'–CCTTGTAGTTGAGAACCAGG–3' and <anti-sense> 5'–GGGCTTGGTATATATGTGG–3'; and for GAPDH were: <sense> 5'–GAAGGTGAAGGTCGGAGTC–3', <anti-sense> 5'–GAAGATGG TGATGGGATTTC–3'. Primers were synthesized at Midland Certified Reagent Company (Midland, TX). The levels of spliced XBP-1 mRNA were measured by using iQ$^{TM}$ SYBR Green Supermix (Bio-Rad) and amplification reactions were performed using the C1000$^{TM}$ Thermal-cycler (CFX96; Bio-Rad). Following cycling conditions were utilized: priming at 95ºC for 5 min, and then 40 cycles of 95ºC for 30 sec, 55ºC for 30 sec, and 72ºC for 30 sec. Fold changes in XBP-1 mRNA expression (ΔCt values) were calculated after normalization to corresponding GAPDH mRNA levels.
